# Disaster preparedness knowledge and its relationship with triage decision-making among hospital and pre-hospital emergency nurses - Ardabil, Iran

**DOI:** 10.1186/s12913-022-08311-9

**Published:** 2022-07-20

**Authors:** Islam Azizpour, Saeid Mehri, Aghil Habibi Soola

**Affiliations:** 1 Department of Emergency nursing, School of Nursing and Midwifery, Ardabil University of Medical Sciences, Ardabil, Iran; 2 Department of Nursing, School of Nursing and Midwifery, Ardabil University of Medical Sciences, Ardabil, Iran

**Keywords:** Disaster preparedness, Triage decisions, Hospital emergency nurses, Pre-hospital emergency nurses

## Abstract

**Background:**

Hospital and pre-hospital emergency nurses are at the forefront of disaster response. Disaster incidents continue to pose a threat to healthcare systems by exposing them to an overwhelming surge of patients.

**Methods:**

This descriptive cross-sectional study was performed on 472 hospital and pre-hospital emergency nurses in Ardabil province, in the northwest Iran, from March to April 2021, were recruited by convenience sampling method. Data were collected using valid and reliable self-reported questionnaires, including the Emergency Preparedness Information questionnaire (EPIQ) and Triage Decision-making Inventory (TDMI). Data were analyzed using SPSS (Version 22) software using descriptive statistics, Pearson correlation coefficient test, t-test, ANOVA test, and multiple linear regression analysis.

**Results:**

Emergency nurses’ disaster preparedness knowledge was low according to the mean score of total disaster preparedness knowledge. Furthermore, multiple linear regression analysis showed triage decision-making, age, residence, disaster preparedness training, working on duty during a disaster, and training organization variables were predictors of disaster preparedness knowledge in hospital and pre-hospital emergency nurses (*p* < 0.05).

**Conclusion:**

Emergency nurses who have higher disaster preparedness knowledge have higher triage decision-making skills. It is suggested that the managers of educational and medical centres and professional organizations provide favourable conditions for training and increasing disaster preparedness of emergency nurses according to their age and residence.

## Introduction

Disasters are situations or events that overwhelm local capacities and require the application of foreign assistance nationally or internationally. A disaster is an unpredictable and often sudden event that causes great damage, destruction, and human suffering. According to statistics provided by the Center for Research on The Epidemiology of Disaster (CRED) in 2019, 396 natural disasters in the world are close to life. The death toll has risen to 11,755, affecting more than 95 million people and costing the economy 130 billion dollars [1].

Health care workers working in various service sectors are one of the vital assets of any country in times of disaster [2] who play a vital role in the implementation of disaster plans [3, 4]. The preparedness and knowledge of frontline respondents, such as emergency nurses, are essential to providing quality care and minimizing additional complications and mortality [5].

In the event of a disaster, emergency nurses, including hospital and pre-hospital emergency nurses, are the first group of healthcare providers to treat the injured and provide the necessary medical care to the disaster victims [2, 3, 6]. The pre-hospital emergency nurses are the first responders in emergencies, from disasters to minor injuries and illnesses [7]. The roles of pre-hospital emergency nurses during disasters include responding to critical situations, detecting danger, reducing or eliminating injuries, managing incidents on the scene, managing and distributing medical equipment, and performing mass care, triage, and emergency treatments [8].

On the other hand, hospital emergency nurses are among the first-line healthcare professionals in the emergency departments that respond to disasters. They make up a large group of those receiving, assessing, and treating victims of disasters [5]. Research conducted to survey this group’s level of preparedness over the past decade reveals low to moderate levels of existing knowledge and perceived preparedness in the area of disaster response [4, 9–11].

Preparedness is the most important step in the Disaster Risk Management (DRM) process [8, 12] based on international documents such as the Sendai Framework for Disaster Risk Reduction (SFDRR) 2015–2030 [8], and includes steps that are already taken to ensure an effective response [12]. Among the various variables in DRM, the health system as the most important organization can prepare appropriate programs and strategies to reduce social and financial losses [12]. In developed countries, health systems need to plan for disaster preparedness and skill development, and this plan must be up-to-date and all personnel must be aware of it [12]. Preparedness of health systems, especially pre-hospital and hospital systems, is essential [8, 12]. Having preparedness for disasters and the proper response of health teams can maintain stability in the community [13].

Disaster preparedness knowledge is the ability to define a disastrous event, understand the incident command system, triage, and assessment and their role in a disastrous event [14]. Emergency nurses must be equipped with the knowledge needed to deal with a disaster because disaster preparedness knowledge can help maintain stability in emergencies [15]. In addition to the above basic understanding of disaster preparedness, public health workers, especially emergency nurses, should be aware of the concept of disaster preparedness and knowledge of infection control, contingency planning to prevent further damage, triage, mass immunization, mass evacuation, and treatments for mass casualties [14].

There are more questions than answers when the disaster preparedness of the nursing profession is considered as nurses play a central role in the disaster response [14]. Yet, research has revealed that most registered nurses are not confident in their abilities to respond to major disaster events [14, 16]. Numerous factors affect the knowledge of disaster preparedness for emergency nurses. At the global level, the existing literature shows the experience of disaster response and education/training in disasters as two factors that have the strongest and most consistent relationship with increasing the level of objective and mental preparedness of nurses [3, 17, 18]. Residence, work experience, years as an emergency nurse [14, 16, 19], age [20], level of education [21, 22], and place of work [20] are the other factors affecting the knowledge of disaster preparedness.

Further, emergency decision-making plays an important role in the disaster response ability [5]. Knowledgeable nurses can help minimize the harmful effects of disasters and be effective for their communities [23]. Therefore, emergency nurses need to have disaster preparedness knowledge to avoid the severity of the effects [24]. In other words, secondary disasters may occur if emergency nurses fail to act as the first responder in the disaster response phase [25]. An unprepared emergency nurse and incorrect triage decisions lead to loss of resources, delays in treatment, dissatisfaction, and adverse consequences [26].

Disasters always have a negative impact on the general health and well-being of the affected population, and efficient health care is a key factor in the survival of most people [27]. The flow of casualties can be maintained through sorting and triage [28]. Triage in emergencies and disasters is a stressful task and one of the major challenges in these situations [29]. During disasters, triage has different goals and processes than triage under normal circumstances. In times of disasters, due to a large number of injured patients and the limited facilities and services, there is a need to properly classify and allocate the best services to the most injured victims [27, 30].

Triage Decision-Making (TDM) is one of the first clinical decisions when caring for a patient. The purpose of triage is to identify the severity of the injury and reduce the negative consequences through rapid evaluation [14, 31]. Decision-making is the most sensitive level of triage, and emergency nurses’ knowledge can be a more effective factor than their performance and experience [32, 33]. Triage decisions are often made quickly, independently, and under time constraints, so they can have a serious impact on patient outcomes and flow. Professional capabilities play an important part in emergency nurses’ decision-making [34]. The literature has consistently shown that critical thinking, cognitive skills, experience, and intuition are key elements in maintaining the accuracy and speed of triage decision-making and thus patient safety [11, 35, 36]. Also, nursing research suggests that TDM has a positive influence on the patient care outcomes [14, 31]. Due to the need for rapid clinical evaluation of patients during disasters, triage decisions may be related to and affect the preparedness of emergency nurses in disasters [14].

Hospital and pre-hospital emergency nurses play an important role in disaster response. A review of the literature shows a wide range of nursing functions, roles, training, education, and background; however, no study has concluded that nurses are fully or even adequately prepared with all the necessary knowledge on disaster preparedness [3, 8, 10, 11, 37–40], Moreover, few studies, such as the Schneider’s 2019 study [14], have focused on the relationship between everyday elements such as triage decisions by disaster-prepared nurses.

A disaster event challenges the resources and support available to respondents and during a disaster, health care facilities in an affected area can fall into a functional decline [13, 14]. Considering the limited resources of nurses during disasters, it is important to understand factors that affect their ability to cope with disasters. Using the results of such studies, appropriate steps can be taken in educational planning and the educational needs of nurses and nursing students by identifying the factors affecting disaster preparedness knowledge. Furthermore, to respond effectively to unforeseen events such as disasters, suggestions can be made to the managers of emergency medical and hospital centers regarding the proper planning of the centers under their management. This study was conducted to determine the level of “disaster preparedness knowledge” and its relationship with “triage decision-making” among hospital and pre-hospital emergency nurses in Ardabil province-Iran.

## Methods

### Study design

This descriptive-analytical study was performed from March to April 2021 in Ardabil province, north-western Iran. Ardabil province has a cold mountainous climate and is famous for its hot springs. Sabalan Mountain is located at an altitude of 4811 m in this province.

### Participants and setting

The statistical population of this study consisted of 472 hospital and pre-hospital emergency nurses, who were selected by convenience sampling method, from 12 educational-medical centers and 9 emergency medical services centers affiliated to Ardabil University of Medical Sciences. Emergency nurses in Iran are composed of hospital and pre-hospital emergency nurses. Pre-hospital emergency nurses have an associate or bachelor’s degree in emergency care. Also, nurses who have a bachelor’s or a master’s degree may be employed in pre-hospital emergencies [34]. The inclusion criteria of the study consisted of having at least an associate degree in nursing education, having been working for a minimum of 6 months in the Emergency Department (ED)/Emergency Medical Services (EMS), and being active during the data collection stage. All those who were on leave during the study time, non-nursing emergency staff, and incomplete questionnaires were excluded from the research. Out of 821 nurses who met the inclusion criteria, 218 did not consent to participate in the study, 89 questionnaires were not returned, and 42 questionnaires were incomplete. Finally, 472 people were included in the study after completing the research participation form.

#### Data collection

Data were collected by an Emergency Preparedness Information Questionnaire (EPIQ) and Triage Decision-Making Inventory (TDMI).

### Emergency preparedness information questionnaire (EPIQ)

EPIQ is a 44-item instrument that was developed by Wisniewski et al. in 2004 [41] and has been used in several studies [19, 37–39]. The questionnaire consists of two parts. The first part with 14 items is about participants’ personal and work-related characteristics such as age, gender, level of education, workplace (hospital and pre-hospital emergency), years of experience, years working as an emergency nurse, marital status, shift type, training in disaster preparedness, caring during a disaster, worked during a disaster, triage training, the organization in charge of training disaster preparation, and residence. The second part assesses the disaster preparedness knowledge of nurses with 8 competency dimensions (items) of emergency preparedness, including emergency nurses’ familiarity with subcategories like incident command system (7 items), triage (6 items), communications (7 items), psychological issues and vulnerable populations (6 items), isolation, quarantine and decontamination (5 items), epidemiology and clinical decision-making (4 items), ability to access critical resources and reporting (4 items), familiarity with biological agents (4 items), and knowledge of understanding preparedness during critical situations (1 item).

The questionnaire is scored based on a 5-point Likert scale (from 1 = Unfamiliar to 5 = Very familiar). The EPIQ in Iran has been translated and evaluated psychometrically by Hassankhani et al. In Hassankhani’s study, the accuracy of the translation of this questionnaire was confirmed by an English language expert and the validity of the instrument in terms of content and face validity was confirmed by a group of 10 university professors. The reliability (internal consistency) of the instrument was calculated by Cronbach’s alpha (α = 0.98). Also, the test-retest method was used to assess the reliability of the questionnaire. Nine questionnaires were distributed among the hospital staff at the same time interval, and the answers were matched. The test-retest index was determined to be *r* = 0. 98, which indicated the compatibility of the questionnaire [37]. Cronbach’s alpha coefficient of the above-mentioned 8 dimensions ranged from 0.83 to 0.94 ^41^ in the original study and from 0.86 to 0.90 in the present study. Cronbach’s alpha coefficient for the whole questionnaire was reported to be 0.98 in Hassankhani et Al’s study [37] and 0.97 in the present study.

### Triage decision-making inventory (TDMI)

TDMI was developed by Cone in 2000 to quantitatively assess the level of self-confidence of emergency department nurses in triage decision-making [31]. The original questionnaire consists of 37 questions and 4 subscales, which was reduced to 27 questions with three subscales in a subsequent study [41].

In this study, a 27-item questionnaire was used, which included cognitive abilities (14 items), experience (6 items), and intuition (7 items). The questionnaire is scored based on a 6-point Likert scale (from 1 = strongly disagree to 6 = strongly agree). The whole score of this questionnaire is 162 [41]. The whole score of this questionnaire and its subscales is obtained by dividing the sum of the scores given by the participants by the total number of questions [36]. After obtaining permission from the original designer, the English version of this questionnaire was translated into Persian by an independent translator. To determine the content validity index and ratio, a questionnaire was given to 12 faculty members of Ardabil University of Medical Sciences (Faculty of Nursing and Midwifery). The Content Validity Index (CVI) was evaluated separately by experts using three criteria of simplicity, appropriateness, and certainty based on a four-part spectrum (for example, in terms of simplicity, quite simple, somewhat complex, and complex) for each question. Relevant ratings were given. Finally, the content validity index was 0.90. Cronbach’s alpha coefficient (α = 0.97) also showed the reliability of the questionnaire was acceptable. Moreover, the test-retest method was used to assess the reliability of the questionnaire. Twenty questionnaires were distributed among the hospital and pre-hospital emergency nurses at the same time interval, then the agreement of the answers was evaluated, and a coefficient of *r* = 0.94 was obtained, which indicates the compatibility of the questionnaire. The researcher confirmed that participants understood each item in the questionnaire during the pilot study; therefore, there were no changes in the items of the questionnaire. In the main study, Cronbach’s alpha ranged from 0.85 to 0.92 for each subscale [41]. In the current study, Cronbach’s alpha for each subscale ranged from 0.80 to 0.94, and it was 0.94 for the whole questionnaire.

### Data analysis

After collecting the questionnaires, the data were analysed using SPSS-ver. 22. At first, data related to demographic and occupational characteristics, disaster preparedness training, caring during a disaster, working on duty during a disaster, and triage training were reported using percentages, means, and standard deviations. Second, the independent t-test was used to investigate the relationship between emergency preparedness information and sex, marital status, shift type, workplace, disaster preparedness training, caring during a disaster, working during a disaster, and triage training. Further, the ANOVA test was used to investigate the relationship between disaster preparedness knowledge and work experience, years working as an emergency nurse, level of education, and passing special disaster management and care courses. Pearson correlation analysis was used to investigate the relationship among disaster preparedness knowledge, triage decision-making, and triage decision-making inventory subscales. Factors affecting disaster preparedness knowledge were identified as predictors via multiple linear regression analysis. Collinearity was controlled with the VIF index. Kolmogorov-Smirnov test was used to evaluate the normality of the data. The results of the normality test showed that the studied data were normal.

## Results

Overall, 472 hospital and pre-hospital emergency nurses participated in this research. The response rate to the questionnaires was 57.5%. The mean ± SD scores of participants’ age, work experience, and years working as emergency nurses were 32.52 ± 6.08 years, 8.1 ± 5.63, and 6.1 ± 4.74, respectively. The majority of participants were male (52.3%), had a bachelor’s degree (85%), were married (65.5%), had rotating shifts (87.5%), were hospital emergency nurses (67.8%), and came from Ardabil province (69.3%). Moreover, 78% of the participants had received disaster preparedness training, and 83.3% had received triage training. In addition, 75.4% of participants had work experience, and 85.5% had patient care experience in times of disasters. Further, 68.4% of the participants had received disaster preparedness training, and 69.3% had received triage training in the hospital setting. The demographic characteristics of study participants and statistical analyses are shown in Table 1.

**Table 1:** Demographic characteristics of emergency nursing staff (*N* = 472)

	Variables	N	%	Mean ± SD	***P*** value
**Age**^**a**^ (32.52 ± 6.08)				2.95 ± .710	*r* = 0.700 *p* = 0.018
**Gender**^**b**^	Male	247	52.3	3.01 ± .695	t = 1.912 *p* = 0.057
	Female	225	47.7	2.88 ± .722	
**Marital Status**^**b**^	Single	163	34.5	2.99 ± .748	t = 0.898 *P* = 0.370
	Married	309	65.5	2.93 ± .689	
**Years of experience**^**c**^	≤5 years	194	41.1	2.94 ± .719	*F* = 0.657 *P* = 0.622
	6–10 years	132	28.0	2.97 ± .706	
	11–15 years	89	18.9	2.87 ± .659	
	16–20 years	44	9.3	3.03 ± .644	
	> 20 years	13	2.8	3.13 ± 1.12	
**Years working as an emergency nurse**^**c**^	≤5 years	270	57.2	2.90 ± .702	*F* = 4.174 *P* = 0.002
	6–10 years	123	26.1	3.08 ± .683	
	11–15 years	56	11.9	2.78 ± .709	
	16–20 years	16	3.4	2.95 ± .555	
	> 20 years	7	1.5	3.69 ± 1.13	
**Shift type**^**b**^	Fixed work shift rotating shifts	59	12.5	3.02 ± .765	t = 0.810 *P* = 0.418
		413	87.5	2.94 ± .702	
**Level of Educational**^**c**^	Associate degree	48	10.2	2.93 ± .645	*F* = 0.237 *p* = 0.789
	Bachelor	401	85.00	2.96 ± .716	
	Master or PhD	23	4.8	2.86 ± .756	
**Workplace**^**b**^	ED^d^	320	67.8	2.89 ± .706	t = − 2.572 *p* = 0.010
	EMS^e^	152	32.2	3.07 ± .706	
**Disaster preparedness training**^**b**^	Yes	368	75.00	3.03 ± .689	t = 4.759 *p* = 0.000
	No	104	22.00	2.66 ± .712	
**Caring during a disaster**^**b**^	Yes	405	85/8	2.99 ± .705	t = 2.928 *p* = 0.004
	No	67	14.2	2.71 ± .700	
**worked during a disaster**^**b**^	Yes	356	75.4	3.02 ± .699	t = 4.052 *p* = 0.000
	No	116	24.6	2.72 ± .699	
**Passing special management or care courses in disaster**^**c**^	No course	106	22.5	2.68 ± .725	*f* = 8.208
	Hospital disaster Management Course	80	16.9	3.09 ± .598	*p* = 0.000
	In-service training	198	41.9	2.95 ± .730	
	disaster Workshop	88	18.6	3.12 ± .654	
**Triage Training**^**b**^	Yes	393	83.3	2.98 ± .690	t = 2.457
	No	79	16.7	2.77 ± .783	*p* = 0.014
**Residence**^**b**^	Center of province	327	69.3	2.96 ± .672	t = 0.568
	Countryside	145	30.7	2.92 ± .791	*p* = 0.033

^a^correlation test

^b^T-test

^c^ANOVA

^d^Emergency Department

^e^Emergency Medical Services

The mean ± SD score of perceived knowledge of all emergency preparedness information questions was 2.95 ± 0.71. The highest and the lowest mean ± SD scores for the subscales were related to triage (3.77 ± 0.45) and biological agents (2.56 ± 0.95) (Table 2).

**Table 2:** Mean of the scores and Coefficient α obtained from disaster preparedness information (*N* = 472)

Subsets	Mean ± Standard Deviation	Coefficient α
Incident command system	3.07 ± 0.84	0.90
Triage	3.45 ± 0.77	0.87
Communication and connectivity	2.95 ± 0.83	0.89
Vulnerable population and psychological problems	2.94 ± 0.83	0.89
Isolation, quarantine, and decontamination	2.76 ± 0.92	0.90
Accessing critical resources and reporting	2.81 ± 0.93	0.87
Epidemiology and clinical decision making	2.79 ± 0.89	0.86
Biological agent detection	2.56 ± 0.95	0.90
Self-reported overall familiarity	2.80 ± 0.95	^a^
Calculated overall familiarity	2.95 ± 0.71	0.97

The (^a^) indicates single response item

The results of the t-test and ANOVA showed a significant relationship between disaster preparedness knowledge and years working as an emergency nurse (*f* = 4.174, *p* = 0.002), workplace (t = 2.572, *p* = 0.010), disaster preparedness training (t = 4.759, *p* = 0.000), caring during a disaster (t = 2.928, *p* = 0.004), working on duty during a disaster (t = 4.052, *p* = 0.000), passing special management or care courses on disaster (*f* = 8.208, *p* = 0.000), triage training (t = 2.457, *p* = 0.014), and residence (t = 0.568, *p* = 0.033) (Table 1).

The mean ± SD score of triage decision-making was 117.71 ± 19.57. Moreover, the mean ± SD scores of triage decision-making subscales (cognitive abilities, intuition, and experience) were reported to be 63.86 ± 11.33, 27.91 ± 6.44, and 25.94 ± 4.94, respectively (Table 3).

**Table 3:** Descriptive statistics and Correlations of the study variables (*N* = 472)

Variable	Mean ± SD	1	2	3	4	5
**Total EPIQ**	2.95 ± 0.71	1.00				
**Total TDMI**	117.71 ± 19.57	0.532a	1.00			
Cognitive Abilities TDMI	63.86 ± 11.33	0.453^a^	0.931^a^	1.00		
Intuition TDMI	27.91 ± 6.44	0.437^a^	0.727^a^	0.467^a^	1.00	
Experience TDMI	25.94 ± 4.97	0.493a	0.869^a^	0.777^a^	0.501^a^	1.00

1 = Total EPIQ

2 = Total TDMI

3 = Cognitive Abilities TDMI

4 = Intuition TDMI

5 = Experience TDMI

^a^Correlation is significant at the 0.01 level

Pearson correlation test was used to test the relationship among disaster preparedness knowledge, triage decision-making, and triage decision-making inventory subscales. There was a significantly positive relationship between disaster preparedness knowledge and triage decision–making and triage decision subscales (*r* = 532, *p* = 000, *N* = 472) (Table 3).

Multiple linear regression predicts factors that affect the preparedness knowledge of hospital and pre-hospital emergency nurses. Triage decision-making subscales (cognitive abilities, intuition, and experience), training organization, age, residence, previous disaster preparedness training, and previous work experience during the disaster at the hospital or emergency centers predict disaster preparedness knowledge when other variables are controlled and also explain 36% of the variance of these competencies. Multiple linear regression showed that among the triage decision-making subscales, experience (β = 0.278, *p* = 0.000) had the highest impact, followed by intuition (β = 0.215, *p* = 0.000) and cognitive abilities (β = 0.128, *p* = 0.040). Besides, there was a positive relationship between training organization (β = 0.131, *p* = 0.030) and disaster preparedness knowledge. There was a significant relationship between disaster preparedness knowledge and residence (β = − 0.093, *p* = 0.020), previous disaster preparedness training (β = − 0.199, *p* = 0.009), and previous work experience in the event of disaster in a hospital or pre-hospital emergency (β = − 0.099, *p* = 0.014). However, there was a significant and negative relationship between disaster preparedness knowledge and age (β = − 0.137, *p* = 0.021) (Table 4).

**Table 4:** Multiple linear regression predicting disaster Preparedness knowledge (*N* = 472)

Variables	B	Std. Error	Beta	T	Sig
Cognitive Abilities TDMI	0.008	0.004	0.128	2.058	0.040
Intuition TDMI	0.024	0.005	0.215	4.867	0.000
Experience TDMI	0.040	0.009	0.278	4.431	0.000
Gender	0.083	0.064	−0.058	−1.294	0.196
Age	0.016	0.007	−0.137	− 2.312	0.021
Level of Education	0.083	0.072	0.047	1.157	0.248
Years working as an emergency nurse	0.011	0.009	0.071	1.193	0.233
Workplace	0.003	0.006	−0.027	− 0.530	0.596
Residence	0.039	0.017	−0.093	−2.330	0.020
Triage training	0.157	0.101	0.083	1.553	0.121
Training organization	0.86	0.039	0.131	2.175	0.030
Passing special management or care courses in disaster preparedness	0.040	0.030	0.058	1.318	0.188
Training in disaster preparedness	0.204	0.077	−0.199	−2.466	0.009
Worked during a disaster	0.163	0.066	−0.099	−2.466	0.014

*R* = 0.600

*R* square = 0.360

*F* = 18.38

*p* = 0.000

## Discussion

Disaster preparedness knowledge is essential for emergency nurses [4, 10]. The World Health Organization (WHO) recommends that all countries, regardless of how often they suffer from disasters, prepare their healthcare professionals to respond to disasters [23]. This study aimed to determine the level of “disaster preparedness knowledge” and its relationship with “triage decision making” in hospital and pre-hospital emergency nurses in Ardabil province.

The results showed that emergency nurses did not have good disaster preparedness knowledge, which is consistent with the results of previous studies [16, 37–40, 42], indicating that frontline health workers need to improve their disaster preparedness knowledge. Also, the findings of Al-Zahrani et al. indicated that nurses had poor knowledge of emergency and disaster preparedness programs [11]. Further, the results of previous studies show that nurses with poor disaster preparedness knowledge can do more harm to disaster victims than help [16, 40]. The average total familiarity of nurses in the eight subscales of disaster preparedness knowledge was low to moderate. Therefore, it is recommended to provide appropriate disaster preparedness information training to emergency nurses to increase their level of preparation.

The results of the present study also demonstrated that the highest level of EPIQ was related to the triage dimension (3.45). The results of other studies also confirm this finding [4, 10, 37, 38]. The high level of familiarity with triage can be because triage concepts have traditionally been included in nursing education programs [43] and emergency nurses have good information about it because it is performed daily in Emergency Departments (EDs) and Emergency Medical Services (EMS) [10].

Moreover, the findings indicated that the lowest level of EPIQ was related to the biological dimension (2.56), which is line with the results of previous reports [17, 23, 44] indicating that the nurses have limited knowledge about biological agents. Considering the predicted increase in natural disasters, the prevalence of new diseases such as COVID-19, it is important to educate nurses and improve their preparedness for critical situations [38].

The results of this study also showed that having disaster preparedness training and the training organization are predictors of disaster preparedness knowledge. These two factors were significantly correlated with the EPIQ score. Training programs are needed to increase nurses’ knowledge and understanding of their roles and responsibilities to respond effectively to disasters [45]. This finding is congruent with those of Omar Ghazi Baker [40, 46] which showed nurses who received training in disaster management were more prepared to deal with disaster situations than those who did not receive such training. The result of a study in Indonesia showed that disaster training increases nurses’ preparedness to deal with disasters [3]. Also, Mohammad Hamdi Abuadas reported previous disaster education as one of the predictors of disaster preparedness among registered nurses in Saudi Arabia [45]. Disaster preparedness training improves nurses’ self-efficacy and disaster management ability and increases their willingness to respond to a disaster [47]. Some of the preparedness strategies include effective education, mock drill training, annual training, and manoeuvres based on staff needs, and engaging in disaster planning, which is useful in increasing the nurses’ knowledge of healthcare services [10, 40, 48] + 91. Lack of training programs is a major problem that leads to a lack of awareness of health professionals about disaster preparedness [40]. This backwardness can be reduced by helping emergency nurses identify and improve their preparedness for disaster response as a part of undergraduate and continuing education programs [49].

The results of multiple linear regression showed a significant and positive relationship between the dimensions of triage decision-making and disaster preparedness knowledge in emergency nurses. Improving triage decisions among all healthcare providers is essential to improving patient flow and consequently patient outcomes, especially during a disaster [28]. Disaster victims receive their first care from emergency nurses. The more accurately and quickly these professionals act, the lower the potential for casualties and disabilities, and the more people can trust the services they provide. Their success also depends on several factors, one of the most important of which is having good decision-making ability [34].

Triage decision making is related to the components of cognitive ability, experience, and intuition [41]. Emergency nurses should effectively use their capabilities in critical emergencies and take effective clinical actions [32]. In disaster management, decisions are made by intuition or reasoning, which promotes quick and accurate decisions [50]. Knowledge, experience, and clinical skills are essential to making clinical decisions [34]. The results of the study by Schneider showed that triage decision-making had a statistically significant influence on disaster preparedness knowledge [14].

In Triage decision-making, nurses’ sufficient knowledge is a more effective factor than their performance and experience [29]. Nurses should be able to quickly identify priorities during triage using their skills in crowded, noisy, and stressful environments [36]. The decision to prioritize patient care in times of disaster is very important considering the high number of patients. Therefore, an emergency nurse who is skilled in triage decision-making is expected to have higher disaster preparedness knowledge than an emergency nurse who is not proficient in triage decision-making.

The results of the present study showed a significant relationship between disaster preparedness knowledge and work during a disaster, which was consistent with the results of previous studies [3, 5, 39, 40]. Chegini et al. found that emergency nurses with previous disaster response experience had significant levels of disaster core competencies compared to those with limited disaster response experience [15] Emaliyawati et al. reported nurses who have never volunteered in a disaster situation have a lower level of disaster preparedness [39]. Nurses from disaster-prone communities can gain experience by volunteering to practice. Field training can help emergency nurses gain valuable experience in disaster preparedness [11] According to empirical learning theory, the clinical learning process requires experience [3]. Creating opportunities for emergency nurses to volunteer in disaster conditions can help them improve their abilities and the quality of the services they provide in such conditions.

Furthermore, the results of the current research showed a relationship between residence and disaster preparedness knowledge, which is inconsistent with the results of a study by Spice, in which residence had no significant relationship with emergency preparedness information [19]. As a person’s length of stay in a city increases, so does the experience a person gains in assessing their preparedness and preparedness for disasters [51]. This result can be attributed to the fact that there are more resources and facilities as well as easy access to educational organizations in the center of the province than in other cities in the province.

The results also showed a significant and negative relationship between age and disaster preparedness knowledge so that disaster preparedness knowledge decreased in nurses with age increase. This finding is consistent with those of Hodge Angela J. et al., [20] showing that nurses were less likely to become familiar with emergency preparedness as their age increased. In another study, Öztekin et al. showed a significant and negative relationship between the clinical competency and age in Japanese nurses [22]. This can confirm that the knowledge of emergency nurses in disaster preparedness has not been updated, and they do not have consistent and serious study in this regard. Older nurses are usually married and have the responsibility of caring for their families in addition to their job. Moreover, such cases make them not have the necessary time and energy to participate in training courses, which can be one of the reasons for the decrease in disaster preparedness knowledge as the age increases.

The strength of this study is the diversity of participants from different sections of the health system, including pre-hospital and hospital, and the large number of samples. The present study had some limitations. The sample was regional and may not be generalizable beyond nurses of similar geographic locations and cultural practices. Furthermore, considering the special requirements of the nursing profession and fatigue, workload, and time constraints in completing the questionnaires during the COVID-19 outbreak, it is recommended to conduct a similar study after complete control of the disease and achieving a suitable sample.

## Conclusion

Triage decision-making is an important skill for emergency nurses during disasters because triage effectively prioritizes disaster victims. The relationship between disaster preparedness knowledge and triage decision-making shows that emergency nurses who have a higher level of triage decision-making skills have more disaster preparedness. Receiving disaster preparedness training, training organization, previous experience of participating in disaster, residence, and age are associated with preparedness knowledge. Two specific methods are suggest to address this issue, including offering nurses routine disaster-related training that keeps them working as volunteers in various disaster conditions and providing effective disaster-related information via seminars, practice and manoeuvre, flyers, and other media. Thus, it is suggested that hospital and EMS administrators and professional organizations understand the importance of disaster preparedness knowledge and provide disaster preparedness training programs to emergency nurses considering their age and residence so that they gain more knowledge in this regard.

## Data Availability

The datasets used and/or analysed during the current study available from the corresponding author on reasonable request.
